# IFIANet: Frequency Attention Network for Time–Frequency in sEMG-Based Motion Intent Recognition

**DOI:** 10.3390/s26010169

**Published:** 2025-12-26

**Authors:** Gang Zheng, Jiankai Lin, Jiawei Zhang, Heming Jia, Jiayang Tang, Longtao Shi

**Affiliations:** 1 College of Computer and Control Engineering, Northeast Forestry Univeristy, Harbin 150040, China2024112776@nefu.edu.cn (J.L.);; 2 School of Information Engineering, Sanming University, Sanming 365004, China

**Keywords:** surface electromyography (sEMG), intent recognition, deep learning, robotic exoskeletons, feature fusion

## Abstract

Lower limb exoskeleton systems require accurate recognition of the wearer’s movement intentions prior to action execution in order to achieve natural and smooth human–machine interaction. Surface electromyography (sEMG) signals can reflect neural activation of muscles before movement onset, making them a key physiological source for movement intention recognition. To improve sEMG-based recognition performance, this study proposes an innovative deep learning framework, IFIANet. First, a CNN–TCN-based spatiotemporal feature learning network is constructed, which efficiently models and represents multi-scale temporal–frequency features while effectively reducing model parameter complexity. Second, an IFIA (Frequency-Informed Integration Attention) module is designed to incorporate global frequency information, compensating for frequency components potentially lost during time–frequency transformations, thereby enhancing the discriminability and robustness of temporal–frequency features. Extensive ablation and comparative experiments on the publicly available MyPredict1 dataset demonstrate that the proposed framework maintains stable performance across different prediction times and achieves over 82% average recognition accuracy in within-experiments involving nine participants. The results indicate that IFIANet effectively fuses local temporal–frequency features with global frequency priors, providing an efficient and reliable approach for sEMG-based movement intention recognition and intelligent control of exoskeleton systems.

## 1. Introduction

Lower limb exoskeleton technology has become a core solution for functional reconstruction in individuals with mobility impairments. By integrating sEMG-based motion intention recognition, inertial measurement unit (IMU)-based motion capture, and other sensing technologies, lower limb exoskeletons achieve precise human–robot coordination. This technology provides joint assistance and motion guidance for individuals with post-stroke lower limb dysfunction, spinal cord injuries, and age-related muscle weakness, thereby significantly improving their voluntary motor abilities [1,2,3,4,5,6,7,8]. sEMG signals are bioelectrical signals that record the activity of the neuromuscular system and reflect muscle states and neural control characteristics. By decoding sEMG signals, human lower limb motion intentions—such as level walking, ramp ascent/descent, stair climbing, and turning—can be accurately identified [9]. These signals are acquired non-invasively via surface electrodes, offering low risk and predictive capability for human motion states. Consequently, sEMG plays a crucial role in lower limb exoskeleton systems by capturing biomechanical gait characteristics to help users regain mobility across various terrains [10]. Moreover, sEMG-based motion analysis holds broad applications in sports science, rehabilitation medicine, and human–machine interaction.

Despite notable progress in deep learning-based temporal and time–frequency modeling, two core challenges remain: (1) during time–frequency transformation, some global frequency features are weakened or lost, resulting in incomplete spectral representations; (2) end-to-end network structures often fail to explicitly differentiate the importance of different frequency bands, causing feature redundancy and reduced discriminability. To address these issues, this study proposes a time–frequency feature learning framework, IFIANet, based on band-specific feature extraction and frequency-domain attention fusion. The network architecture is carefully designed to account for the non-stationary nature of sEMG signals. Specifically, the constructed CNN–TCN time–frequency feature learning network employs convolution-based temporal modeling to effectively capture short-term dynamic variations within local temporal segments. At the same time, dilated convolutions are used to build multi-scale temporal receptive fields, enabling the network to simultaneously capture transient changes and relatively long-term temporal dependencies, thereby enhancing its ability to model non-stationary dynamics across multiple temporal scales. Compared with RNN-based models that rely on hidden state recursion, this architecture avoids implicit assumptions of long-term stationarity and provides higher flexibility and parallel computation efficiency when modeling local non-stationary variations. Moreover, continuous wavelet transform (CWT) maps the signal into the time–frequency domain, effectively capturing instantaneous spectral changes over time and thereby modeling non-stationary patterns related to movement states. On this basis, the IFIA module is further introduced, incorporating global frequency priors extracted via fast Fourier transform (FFT) to dynamically adjust the weights and interactions of time–frequency features. This compensates for frequency information loss and enhances feature discriminability and model robustness. Extensive ablation and comparative experiments on the publicly available MyPredict1 dataset [11] demonstrate that the proposed framework outperforms existing methods across multiple gait intention recognition tasks and achieves over 82% average recognition accuracy in experiments involving nine participants. These results validate the effectiveness of IFIANet in time–frequency feature modeling and frequency-domain fusion, providing a novel technical approach and research reference for sEMG-based movement intention recognition and human–machine collaborative control of lower limb exoskeletons.

The main contributions of this study can be summarized as follows:

1. For sEMG-based movement intention recognition, a CNN–TCN-based time–frequency feature learning network is built. The network uses a frequency-partitioned convolution structure to independently extract features from different frequency bands, enabling efficient multi-scale time–frequency modeling and representation.
2. The IFIA module is proposed to perform frequency-guided feature fusion. It uses frequency-domain features extracted via FFT as global guidance to dynamically weight and interact with time–frequency features, strengthening their global discriminability and compensating for potential loss of frequency information during time–frequency transformation.
3. The method is validated on the MyPredict1 dataset, which includes 17 locomotion patterns such as level-ground walking, ramp ascent/descent, stair climbing, and multi-directional small-step movements. Results show high accuracy and stability in lower limb movement intention recognition, with within-subject average accuracy exceeding 92%, demonstrating robustness and generalization.

The rest of this paper is organized as follows: Section 2 discusses the related research on time-domain, frequency-domain, and time–frequency-domain feature extraction and recognition methods of sEMG signals based on machine learning and deep learning; Section 3 details the dataset and data preprocessing methods; Section 4 describes the proposed IFIANet architecture and its key modules; Section 5 presents the experimental results and analysis; Section 6 discusses the model’s performance in addressing existing challenges and outlines future research directions; and Section 7 summarizes the paper.

## 2. Related Work

The performance of sEMG-based movement intention recognition mainly depends on the design of feature extraction and classification strategies [12]. The goal of feature extraction is to highlight discriminative components in the signals while suppressing noise and redundant information, thereby improving the separability of different movement patterns. Over the past decade, various sEMG feature representation methods have been proposed, which can be broadly categorized into time-domain, frequency-domain, and time–frequency-domain approaches [13].

In terms of time-domain analysis, Tkach et al. [14] noted that time-domain features do not require additional signal transformations, offering high computational efficiency and real-time performance. They also proposed a feature selection mechanism based on the stability index to improve within-subject generalization. Frequency-domain features, on the other hand, can more directly characterize the energy distribution of muscle contractions. Kumar et al. [15] performed frequency-domain fusion on multi-channel sEMG signals to effectively capture the frequency energy characteristics of muscle contractions, significantly improving recognition accuracy.

However, traditional time–frequency analysis methods are constrained by the Heisenberg uncertainty principle, which imposes an inherent trade-off between temporal and frequency resolution. Continuous wavelet transform (CWT) can adaptively adjust time and frequency resolution at different scales, enabling multi-scale analysis of non-stationary signals. This allows more detailed capture of transient changes and dynamic frequency components in sEMG signals, providing rich input representations for deep feature learning. Liu et al. [16] applied CWT to transform multi-channel sEMG signals into time–frequency maps, and then used the DIFT-Net to learn their time–frequency features, achieving recognition performance superior to traditional methods. Nevertheless, CWT is still limited by the uncertainty principle, and frequency information may be lost in regions with dense spectral energy or abrupt signal changes, thereby affecting feature completeness and discriminability [17,18].

To overcome the aforementioned limitations, researchers have further introduced deep learning-based feature extraction methods. According to the approach for feature extraction and model training, existing methods can be broadly divided into traditional and deep learning-based approaches. Traditional methods typically rely on manually designed features combined with classical machine learning algorithms, such as linear discriminant analysis (LDA) [19], support vector machines (SVMs) [20,21,22], and random forests (RFs) [23,24]. These methods offer good interpretability and computational efficiency, but their performance is limited in complex gait recognition and within-subject generalization tasks.

In contrast, deep learning methods can automatically learn discriminative features directly from raw signals, avoiding the need for labor-intensive manual feature design. Convolutional neural networks (CNNs) have demonstrated excellent performance in spatial feature extraction [25]. Frank Kulwa et al. [26] applied 2D-CNNs to capture the spatial distribution of multi-channel lower limb sEMG signals, achieving recognition of level-ground walking, downhill walking, and other movement patterns, with a 9% improvement in accuracy compared to traditional SVMs. Recurrent neural networks (RNNs), such as LSTMs, effectively model temporal dependencies [27,28]. Dai Yuanchao et al. [29] used CWT to extract time–frequency features from sEMG signals and combined them with a four-layer LSTM network to evaluate movement correctness, achieving an average F-score improvement of 4.74% over GRU and CNN models. Yanzheng Lu et al. [10] proposed a stacked CNN-LSTM model that extracts time-domain, frequency-domain, and time–frequency features from 8-channel lower limb sEMG signals to estimate hip, knee, and ankle joint angles continuously across seven movement modes, including walking and running, with time-domain features performing best. Since LSTMs are computationally intensive and difficult to parallelize for long sequences, temporal convolutional networks (TCNs) have recently emerged as an efficient alternative. Jiaqing Liu et al. [30] proposed a metric learning-guided ML-TCN, which recognizes four gait phases from six-channel lower limb sEMG signals. The model achieved 96.22% accuracy on complex terrains including level ground, uphill, and downhill, outperforming LSTM (91.20%), and showed 8.15–10.61% higher robustness under 10–50% noise interference. Moreover, the introduction of attention mechanisms has further improved feature selection and discriminability. Attention can adaptively assign importance weights to different time intervals, frequency bands, or channels, emphasizing discriminative features while suppressing redundant information [31]. Zhiwei Zhou et al. [32] proposed the CNN-TL lower limb movement recognition model, integrating self-attention and positional encoding into a CNN-LSTM framework for sEMG signal modeling. In experiments on stair ascent, stair descent, level-ground walking, and squatting, the model achieved 96.13% accuracy, a 3.76% improvement over the baseline CNN-LSTM.

Overall, although existing deep learning models have achieved notable progress in learning time-domain and time–frequency features, they still suffer from limitations in effectively exploiting frequency-domain information. End-to-end training frameworks tend to fit the data in a global manner, making it difficult to explicitly distinguish the relative importance of different frequency components, which may result in feature redundancy and constrain further performance improvements. In contrast, attention-based methods usually operate within a single domain, and such single-domain modeling strategies are insufficient to achieve optimal recognition accuracy. Nevertheless, frequency-domain features encode global discriminative information related to muscle contraction patterns and energy distribution, providing enhanced stability and robustness against noise. Therefore, incorporating frequency-guided mechanisms into deep neural networks to dynamically regulate and enhance discriminative representations during time–frequency feature learning is expected to substantially improve the performance of sEMG-based movement intention recognition.

## 3. Dataset and Data Preprocess

### 3.1. Dataset

The IFIANet was evaluated using the authoritative MyPredict1 database [11], which contains sEMG recordings from ten healthy subjects. However, because the data of one subject were incomplete and included only 14 motion classes, only the remaining nine subjects with complete recordings were used for the analysis. During the experiments, participants walked at a self-selected speed, performing ten consecutive trials followed by a rest period, with each trial lasting approximately 1.5 min. Erroneous samples present in the raw data were removed during preprocessing, resulting in a valid dataset comprising 17 motion intentions, with the corresponding labels listed in Table 1. sEMG signals were collected using Trigno electrodes in a bipolar configuration at a sampling frequency of 1000 Hz. The measured muscles included the gluteus maximus (Gmax), rectus femoris (RF), vastus lateralis (VL), biceps femoris (BF), semitendinosus (ST), tibialis anterior (TA), gastrocnemius medialis (GM), and gluteus medius (Gmed).

### 3.2. Data Preprocess

**Figure 1:**
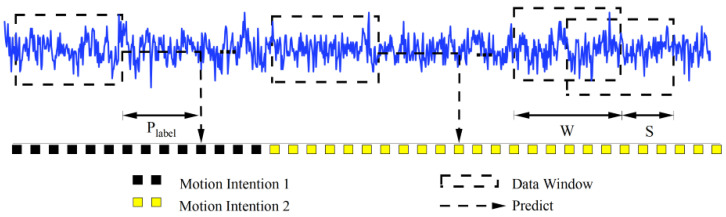
Illustration of data labeling.

**Figure 2:**
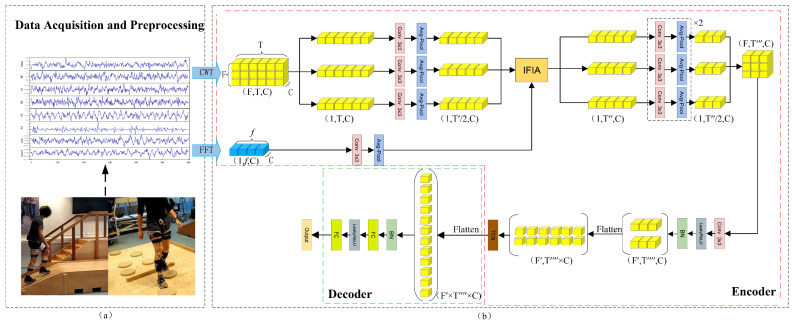
Proposed gait intention recognition framework. (**a**) shows data acquisition and preprocessing, (**b**) shows the encoder–decoder structure with the IFIA module.

In the preprocessing stage, sEMG signals were primarily segmented and normalized. First, the data were segmented using an overlapping sliding window technique [33]. To meet the quasi-real-time intention estimation requirements of lower limb exoskeletons, a window length of 1200 ms and a sliding step of 200 ms were used. The label of each window was determined by the motion event corresponding to the $P_{\mathrm{label}}$ [34] time. The detailed labeling procedure is illustrated in Figure 1, where $P_{\mathrm{label}}$ denotes the prediction time, *W* represents the window length, and *S* indicates the sliding step. Next, noise was removed using a Butterworth high-pass filter with a cutoff frequency of 20 Hz and a sampling rate of 1000 Hz, effectively eliminating low-frequency noise such as motion artifacts and baseline drift while preserving high-frequency components related to muscle activity [35]. To mitigate the influence of amplitude differences across channels and reduce the effect of outliers, each channel was normalized using the 99th percentile of its amplitude. Furthermore, the time-domain signals were transformed into frequency-domain and time–frequency representations using the FFT and CWT, respectively, and subsequently fed into the encoder for feature extraction, as illustrated in Figure 2a.

## 4. Proposed Method

This section provides a detailed description of the proposed sEMG-based gait intention recognition framework, IFIANet, as illustrated in Figure 2b. The framework consists of an encoding module and a decoding module, with the encoder incorporating the IFIA module.

### 4.1. Overall Network Structure

sEMG signals are non-stationary and time-varying bioelectrical signals, whose features can be extracted from the time, frequency, and time–frequency domains. Existing deep learning-based sEMG motion intention recognition models mostly focus on extracting features from either the time domain or the time–frequency domain, while frequency-domain information is typically used only as an auxiliary feature due to its inability to capture the time-varying characteristics of sEMG signals. It is worth noting that frequency-domain features reflect the overall frequency distribution of the signals and thus carry important global discriminative information for distinguishing different motion patterns. Although time–frequency transforms can reveal the dynamic variations of signals along both temporal and frequency dimensions, they may lose some global frequency information during the transformation, resulting in incomplete feature representations. Moreover, different muscle groups often exhibit stable energy distributions within specific frequency bands during contraction, and these frequency components contain global characteristics indicative of motion patterns. However, existing methods fail to effectively leverage frequency-domain features to guide time–frequency feature learning during feature fusion, or only employ static concatenation or simple weighting for frequency-domain integration. This not only limits the discriminative contribution of frequency information but may also introduce redundant features, thereby reducing the model’s ability to distinguish different motions. Therefore, enhancing the global discriminative capability of time–frequency features without introducing redundancy becomes a critical challenge. Cross-attention mechanisms can adaptively model the correlations among different feature components, highlighting complementary information while suppressing redundant responses, thereby enabling efficient coordination and selection of information during feature fusion. Based on these advantages, we propose a frequency-guided deep learning framework for time–frequency feature extraction, as illustrated in Figure 2.

In the proposed time–frequency feature-based deep learning framework, convolution and pooling operations are first applied to both the time–frequency and frequency-domain signals to extract their respective features. After the second convolution and pooling layer in the time–frequency branch, these features are fused with the frequency-domain features via the IFIA module, which adaptively modulates the representations by weighting and compensating for potential loss of global frequency information during the time–frequency transformation. This fusion effectively enhances the discriminability and robustness of the time–frequency features. Subsequently, a temporal convolutional network (TCN) captures temporal dependencies to complete the feature encoding. Finally, a fully connected classification layer decodes the encoded representations to achieve accurate motion intention recognition.

### 4.2. Encoder

The encoder consists of a CNN-based time–frequency feature extraction module, a frequency-domain feature extraction module, a TCN-based temporal modeling module, and the IFIA module based on an attention mechanism. The time–frequency feature extraction module employs a three-stage convolutional network to hierarchically model temporal information representations across different frequency domains, progressively extracting multi-scale discriminative features. After the second convolutional layer, the IFIA module is introduced to achieve guided fusion of time–frequency features with frequency-domain features. To further capture high-level temporal representations and facilitate the transition from convolutional structures to sequential structures, a frequency-domain aggregation convolutional layer is incorporated at the end of the time–frequency feature extraction module. The resulting feature tensor is then rearranged along specific dimensions to enable subsequent temporal modeling.

#### 4.2.1. Convolution Part

In the time–frequency feature extraction module, we draw inspiration from the core concept of depthwise separable convolution [36]. At each convolutional stage, two-dimensional convolutions are applied to signals across different frequency bands, followed by an average pooling layer for downsampling. This design not only effectively reduces the number of parameters and computational cost of the convolutional layers but also enables the extraction of discriminative features from each frequency band while maintaining their independence. To facilitate the transition from convolutional structures to temporal modeling and to integrate the collaborative relationships among multiple frequency bands, the time-domain features are fed into the final convolutional layer, allowing for aggregated modeling of features across different frequency domains. This layer consists of a 2D convolutional layer, a batch normalization (BatchNorm) layer, and a Leaky-ReLU, and the output dimensions are rearranged to (T,F×C). For the frequency-domain feature extraction module, two convolutional layers followed by a max-pooling layer are employed to preserve salient frequency energy distribution features and enhance their discriminative capacity. The specific parameter settings and hierarchical details are provided in Table 2.

#### 4.2.2. TCN Part

To effectively model the temporal dynamics of sEMG signals, we employ a temporal convolutional network (TCN) [37,38] to capture long-range dependencies. Unlike traditional recurrent neural networks (RNNs) such as LSTM, TCN does not rely on sequential outputs from previous time steps; instead, it achieves parallel temporal modeling via dilated convolutions along each channel, significantly improving training efficiency. The TCN module receives an input sequence of length 16. We adopt three stacked Temporal Blocks, each containing two layers of one-dimensional dilated convolution with kernel size 3, followed by BatchNorm and Rectified Linear Unit (ReLU) activation. The dilation rates of the three blocks are set to 1, 2, and 4, respectively. This exponential increase in dilation allows the network to progressively expand its temporal receptive field. For this configuration, the effective receptive field of the three-layer TCN fully covers the input sequence length of 16. Consequently, the TCN is capable of modeling all temporal dependencies within the input window. The channel configuration of the three Temporal Blocks is set to 128, 256, and 128, following an expansion–compression design. Increasing the number of channels in the intermediate block enhances the representational capacity for complex temporal features, while reducing the channels in the final block helps control model complexity and mitigate overfitting. To prevent future information leakage due to convolutional padding, a Chomp operation is introduced to remove excess padding, and residual connections are employed to facilitate gradient propagation. Finally, the extracted temporal features are flattened and fed into the subsequent classification layer.

#### 4.2.3. IFIA

In the time–frequency and frequency feature extraction branches, the former focuses on capturing local time–frequency features, while the latter emphasizes global spectral characteristics. Although these branches provide complementary information to some extent, they may still contain feature redundancy and noise interference. Traditional feature fusion methods, such as element-wise addition or concatenation, lack the ability to selectively weight features, often resulting in accumulated redundancy and reduced discriminative power and robustness.

**Figure 3:**
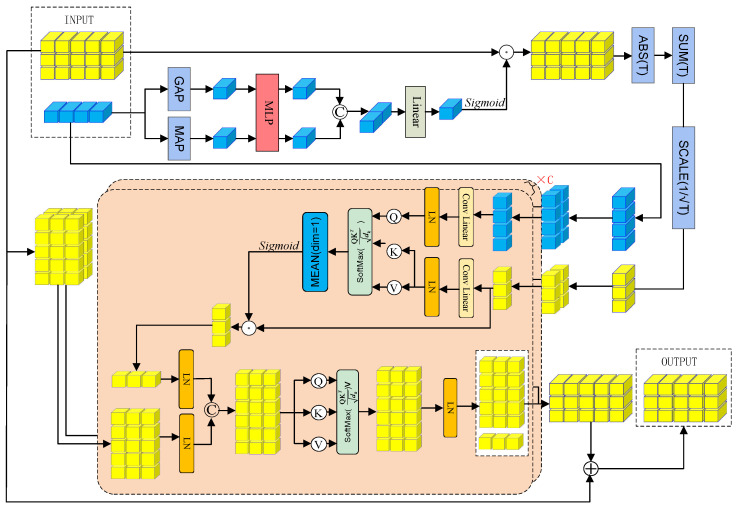
IFIA module.

To address these limitations, we design the IFIA module to achieve adaptive and interactive fusion of frequency and time–frequency features. Its structure is illustrated in Figure 3. The module consists of two stages: channel enhancement and cross-domain interaction. In the channel enhancement stage, frequency-domain features extracted via FFT serve as a global spectral prior, based on which channel-level guidance weights are adaptively generated according to the spectral energy distribution. These weights select and amplify the most representative and relevant time–frequency channels for interaction with frequency features, performing channel-level frequency modulation before entering the cross-domain interaction stage and providing a precise foundation for subsequent feature integration.

During the cross-domain interaction stage, the frequency-guided time–frequency features and the original time–frequency features are jointly fed into a multi-head self-attention structure to model bidirectional dependencies across domains. The guided time–frequency features are temporally compressed to represent the global frequency modulation, while the original features retain their full temporal structure. The multi-head self-attention mechanism dynamically adjusts the response weights at each time step based on the correlation between the two feature sets, selectively enhancing or suppressing local time–frequency features, thereby achieving dynamic optimization of time–frequency representations under the guidance of global spectral information.

This design effectively suppresses cross-domain feature redundancy and noise interference while enabling the model to focus on salient frequency bands and transient regions relevant to motion intention in the time–frequency space, significantly improving the discriminative power and robustness of feature representations. The detailed implementation of the IFIA module is provided in Appendix A.

### 4.3. Decoder

The decoder module is primarily responsible for mapping the high-dimensional time–frequency features extracted by the encoder into the classification space to achieve final motion recognition. Specifically, the feature vectors from the encoder are first normalized using BatchNorm to stabilize the feature distribution. Then, a fully connected layer compresses the high-dimensional features into a low-dimensional embedding space (dim = 32), where a Leaky-ReLU is applied to enhance nonlinear representation capability. Additionally, a dropout layer is introduced to mitigate overfitting. Finally, another fully connected layer maps the embedded features to the category space, generating the final prediction results.

## 5. Experiment Results and Discussion

### 5.1. Experiment Settings

The proposed gait intention recognition model was implemented based on the PyTorch (version 2.6.0) framework. The experimental platform was equipped with an NVIDIA GeForce RTX 3090 GPU with 24 GB of memory, which was used for model training and validation.

To train and evaluate our model, for each subject dataset, all trials were randomly shuffled, and a five-fold cross-validation strategy was adopted to divide the trials into five non-overlapping groups. Within each fold, the trials were split into training, validation, and testing sets with a ratio of 7:1:2. During training, the batch size was set to 128, the number of epochs was 100, and the initial learning rate was 0.003. The AdamW optimizer (*β* = (0.9, 0.999), weight decay = 0.0001) combined with the ReduceLROnPlateau learning rate scheduler was employed to maintain the advantage of adaptive learning while alleviating overfitting [39]. Early stopping was adopted to avoid overfitting. The cross-entropy loss function was used as the loss function:

(1) $$L=-\frac{1}{N}\sum_{n=1}^{N}\sum_{i=1}^{C}y_{i}^{n}\log\left({\hat{y}}_{i}^{n}\right)$$

where *N* is the number of samples in the batch, *C* is the number of classes, $y_{i}^{n}$ is the one-hot encoded true label for the *n*-th sample at class *i*, and ${\hat{y}}_{i}^{n}$ is the predicted probability for class *i* for the *n*-th sample.

### 5.2. Ablation Study

**Figure 4:**
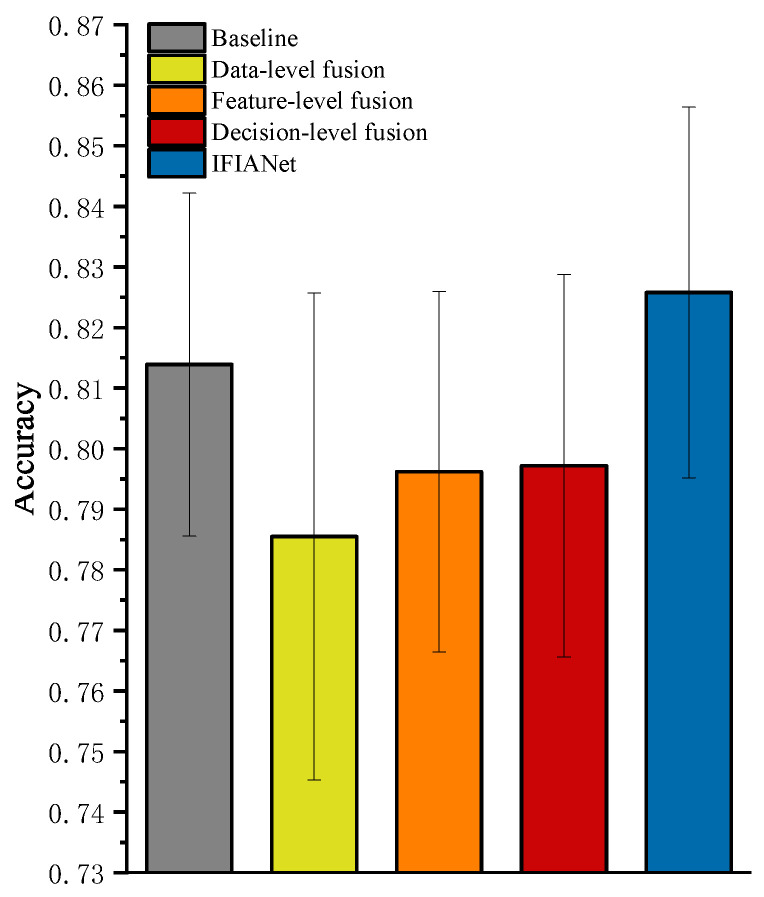
Ablation results of the proposed IFIA.

To compensate for the potential loss of frequency-domain information during the transformation from time-domain to time–frequency representations, we propose an IFIA module based on a CNN–TCN network. This module utilizes frequency-domain information extracted via FFT to guide the fusion of time–frequency features obtained through CWT. Unlike conventional methods that directly concatenate frequency-domain and time–frequency signals or treat them as independent branches for feature or decision fusion, the IFIA module can more effectively leverage frequency-domain information, enabling dynamic guidance and fusion of time–frequency features and thereby enhancing the model’s ability to capture critical features. To validate the effectiveness of the IFIA module, extensive ablation experiments were conducted on a gait recognition dataset under the $P_{\mathrm{label}}$ = 100 ms condition. Using the CNN–TCN network as the baseline, multiple ablation schemes were designed within this framework to evaluate the impact of different modules and fusion strategies on model performance. Three fusion schemes—data-level fusion, feature-level fusion, and decision-level fusion—were constructed. To ensure consistency of the output dimensions across branches at the fusion layer, frequency-domain signals were aligned with time–frequency features via bilinear interpolation. The number of parameters for each method is reported in Table 3 to demonstrate model complexity. The experimental design is illustrated in Figure 4.

The experimental results indicate that the baseline model achieved an accuracy of 81.39±2.83%, with 681,737 parameters, whereas the IFIANet incorporating the IFIA module reached 82.58±3.06%, with 1,087,329 parameters, representing an improvement of 1.19%. Among the three fusion strategies, decision-level fusion performed best, achieving an accuracy of 79.72±3.16% with 1,361,734 parameters. Although slightly higher than that of feature-level fusion, its computational cost is relatively higher. The accuracies of data-level and feature-level fusion were 78.55±4.02% and 79.62±2.98%, respectively, both lower than the baseline. This performance drop may be attributed to the inability of data- and feature-level fusion to establish effective dependencies between frequency-domain and time–frequency features; direct aggregation can lead to information overlap and redundancy between the two, thereby reducing the model performance. In contrast, the proposed IFIA module achieves efficient fusion of multi-scale frequency-domain and time–frequency features with relatively low computational cost, effectively avoiding interference from redundant information while enhancing the response to key features. Among all ablation configurations, the IFIA module consistently exhibits the best performance and shows a clear advantage over conventional fusion methods. This demonstrates that the module can fully leverage global frequency-domain priors to guide and enhance time–frequency representations, thereby achieving a stable improvement in overall recognition performance.

**Figure 5:**
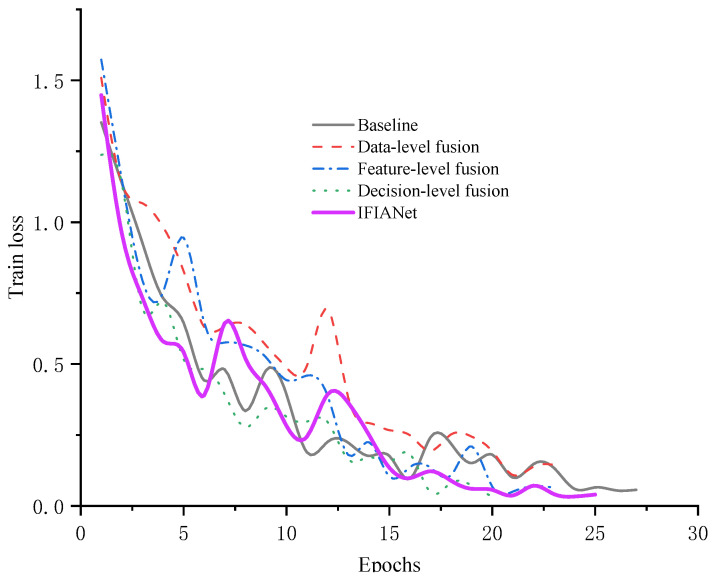
Train loss curves of different ablation configuration schemes.

To analyze the impact of different fusion strategies on model performance, Figure 5 presents the training loss curves under various ablation configurations. As shown, due to the high noise level in the sEMG gait dataset, the baseline model exhibits large fluctuations in loss throughout training and converges unstably. After introducing the proposed IFIA module, the loss curve of IFIANet becomes smoother and achieves early convergence in fewer training epochs. This indicates that the IFIA module can effectively leverage frequency-domain information to suppress noise in time–frequency features, thereby enabling adaptive feature selection and weighted optimization. In contrast, data-level, feature-level, and decision-level fusion models still show noticeable fluctuations in the later stages of training. Although the decision-level fusion model can converge on the training set in approximately 20 epochs, its generalization performance on the test set does not improve significantly. Overall, IFIANet demonstrates the best convergence speed and training stability, further validating the effectiveness and robustness of the IFIA module in feature fusion and noise suppression.

### 5.3. Ablation of IFIA Components

To further analyze the effectiveness of the IFIA module itself, ablation experiments were performed by selectively removing key components of the module. Extensive ablation experiments were carried out on a gait recognition dataset with a single participant under the $P_{\mathrm{label}}$ = 100 ms condition. The IFIA module utilizes frequency-domain information extracted via FFT to guide the dynamic fusion of time–frequency features obtained through CWT, enhancing the network’s ability to capture critical gait features. Three ablation variants were designed: IFIA without channel attention, IFIA without cross-interaction, and IFIA without temporal compression. Comparing the performance of these variants with the full IFIA module allows us to quantify the contribution of each component to the overall model performance.The experimental results are shown in Table 4.

The experimental results show that IFIA without channel attention achieves 80.74%, which is not significantly different from the baseline. This indicates that without channel attention, the model struggles to effectively establish interactions and fuse local frequency components in the time–frequency features with the global frequency information. IFIA without cross-interaction achieves 80.61%, showing a decrease of 0.14% compared to the baseline. This suggests that, without cross-attention, directly weighting the global frequency distribution onto the time–frequency signals may even degrade the model’s performance. IFIA without temporal compression achieves 80.02%, a decrease of 0.73% relative to the baseline. This is mainly because the CWT-based time–frequency features lack compression along the temporal dimension, resulting in a significant scale mismatch between time–frequency and frequency representations. Finally, the proposed model, which incorporates all operations, achieves 81.59%, an improvement of 0.84% over the baseline. This confirms that the collaborative effect of all operations can effectively establish interactions between time–frequency and frequency representations, demonstrating the effectiveness of the IFIA module.

### 5.4. Visualization Experiments of Ablation Study

**Figure 6:**
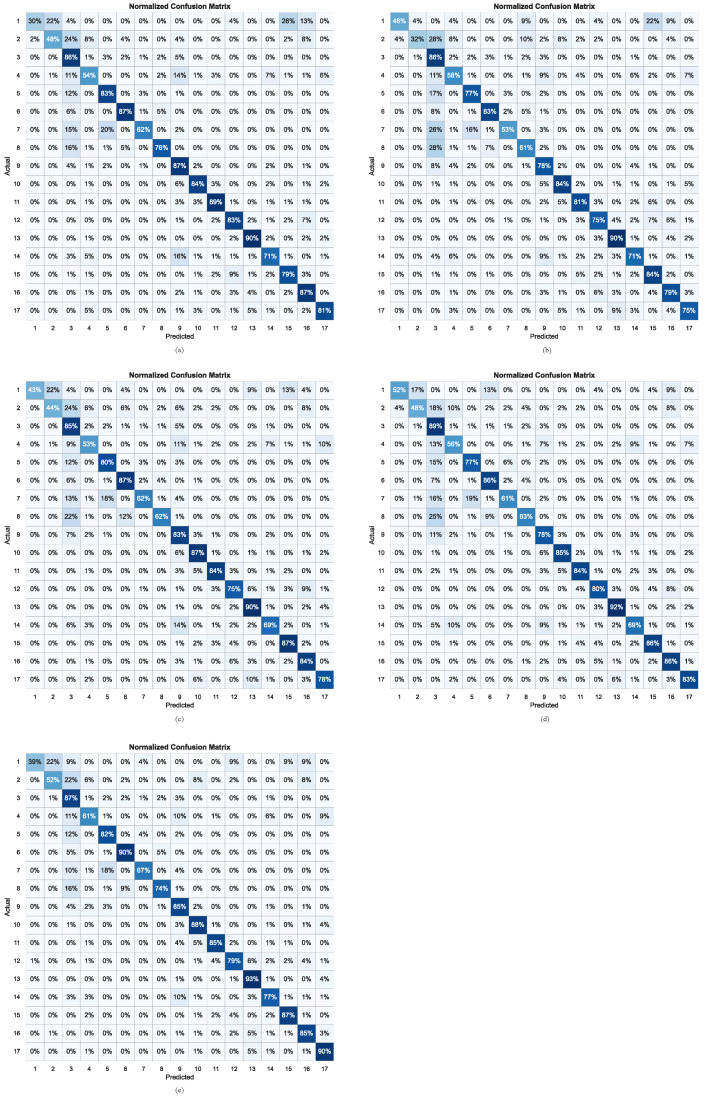
Confusion matrices of different fusion strategies in the ablation study. (**a**) Baseline. (**b**) Data-level fusion. (**c**) Feature-level fusion. (**d**) Decision-level fusion. (**e**) IFIANet.

To further verify the effectiveness of the proposed IFIANet framework, Figure 6 presents the confusion matrices of the different networks shown in Figure 4 for one subject, providing an intuitive visualization of the recognition performance across various motion intentions. The horizontal axis represents the predicted labels, while the vertical axis denotes the ground-truth labels. As shown in Figure 6a, due to the relatively limited number of samples for label 1 (Sitting) and label 2 (Standing), the baseline model fails to effectively capture discriminative features. Specifically, label 1 (Sitting) is frequently misclassified as label 2 (Standing), label 15 (Small steps to the right), and label 16 (Small steps backwards), whereas label 2 (Standing) is mainly misclassified as label 3 (Walking). Meanwhile, label 4 (Turn around axes) is prone to being misclassified as label 3 (Walking) and label 9 (Walking on uneven terrain). The former reflects confusion between static postures, primarily because both sitting and standing exhibit low muscle activation levels, resulting in low-amplitude sEMG signals with limited temporal variation, which makes them difficult to distinguish. The latter represents confusion among dynamic gait patterns, as turning and walking on uneven terrain are both continuous locomotion tasks that often share similar primary driving muscle groups. Moreover, muscle activation patterns in these tasks vary continuously over the gait cycle, leading to substantial overlap in spectral energy distributions and time–frequency representations. In addition, slight electrode displacement or variations in skin–electrode impedance may further increase intra-class variability, thereby masking subtle spectral differences between tasks. Since the baseline model is mainly composed of CNN and TCN modules, although it can capture temporal dynamics, it exhibits limited sensitivity to spectral variations, making it difficult to discriminate gait classes with similar frequency characteristics.

As illustrated in Figure 6e, compared with the baseline model, the introduction of the IFIA module results in significant performance improvements across multiple classes. In particular, the recognition accuracies of label 1 (Sitting), label 4 (Turn around axes), label 14 (Small steps in front), label 15 (Small steps to the right), and label 17 (Small steps to the left) are improved by 9%, 7%, 6%, 8%, and 9%, respectively. These results indicate that the IFIA module can effectively exploit frequency-domain information and enhance the network’s perception of spectral distribution patterns, thereby improving the discrimination between static and dynamic gait states. Consequently, the model exhibits increased robustness under highly imbalanced sample distributions. As shown in Figure 6b, the data-level fusion method partially alleviates the confusion among certain classes; however, the recognition accuracies of label 2 (Standing), label 5 (Ascending stairs), label 7 (Ascending a ramp), label 8 (Descending a ramp), label 9 (Walking on uneven terrain), label 11 (Diagonal step in front right), label 12 (Diagonal step backwards right), label 16 (Small steps backwards), and label 17 (Small steps to the left) still decrease to varying degrees, with reductions of 16%, 6%, 9%, 15%, 9%, 8%, 8%, 8%, and 6%, respectively. This degradation can be attributed to the fact that data-level fusion is unable to effectively embed global frequency priors into local time–frequency features, resulting in limited frequency localization capability and insufficient modeling of dynamic spectral variations. As shown in Figure 6c, the feature-level fusion method leads to decreases in recognition accuracy for label 1 (Sitting), label 8 (Descending a ramp), label 11 (Diagonal step in front right), and label 12 (Diagonal step backwards right) by 13%, 14%, 5%, and 8%, respectively. These results suggest that this approach fails to adequately coordinate time–frequency features with frequency-domain representations, thereby limiting the overall feature modeling capability. As shown in Figure 6d, the decision-level fusion method improves the recognition accuracies of label 1 (Sitting), label 8 (Descending a ramp), and label 15 (Small steps to the right) by 22%, 13%, and 7%, respectively, resulting in an overall enhancement in discriminative performance. However, this method still exhibits noticeable confusion for label 8 (Descending a ramp), with a considerable portion of samples being misclassified as label 3 (Walking).

In summary, the proposed IFIA module effectively integrates global frequency information without disturbing the frequency localization of time–frequency features, thereby enhancing the spectral representation capability of time–frequency features. This leads to significant improvements in recognizing both static and dynamic motion intentions, further validating the superiority and effectiveness of IFIANet in fine-grained spectral modeling and cross-modal recognition tasks.

### 5.5. Effect of MLP Reduction Ratio in IFIA Module

To emphasize more informative global frequency-domain prior features, the proposed IFIA module employs an MLP to highlight the importance of frequency channels. To further investigate the impact of the MLP configuration on the IFIA module, experiments with different values of *r* were conducted to identify the optimal parameter setting. The experimental results corresponding to different reduction ratios are summarized in Table 5. All experiments were conducted on the gait recognition dataset with a single subject under the $P_{\mathrm{label}}$ = 100 ms condition.

As shown in Table 5, the classification accuracy of the IFIANet generally exhibits a decreasing trend as *r* increases. Notably, the highest accuracy of 81.59% is achieved when r=4. When *r* is further increased, the network performance degrades accordingly. This observation indicates that an excessively large reduction ratio may limit the capability of the MLP to model inter-channel dependencies, while a moderate value provides a better trade-off between model complexity and representational capacity. Therefore, r=4 is selected as the optimal reduction ratio for the MLP in the IFIA module.

### 5.6. Comparison of Different Models

**Figure 7:**
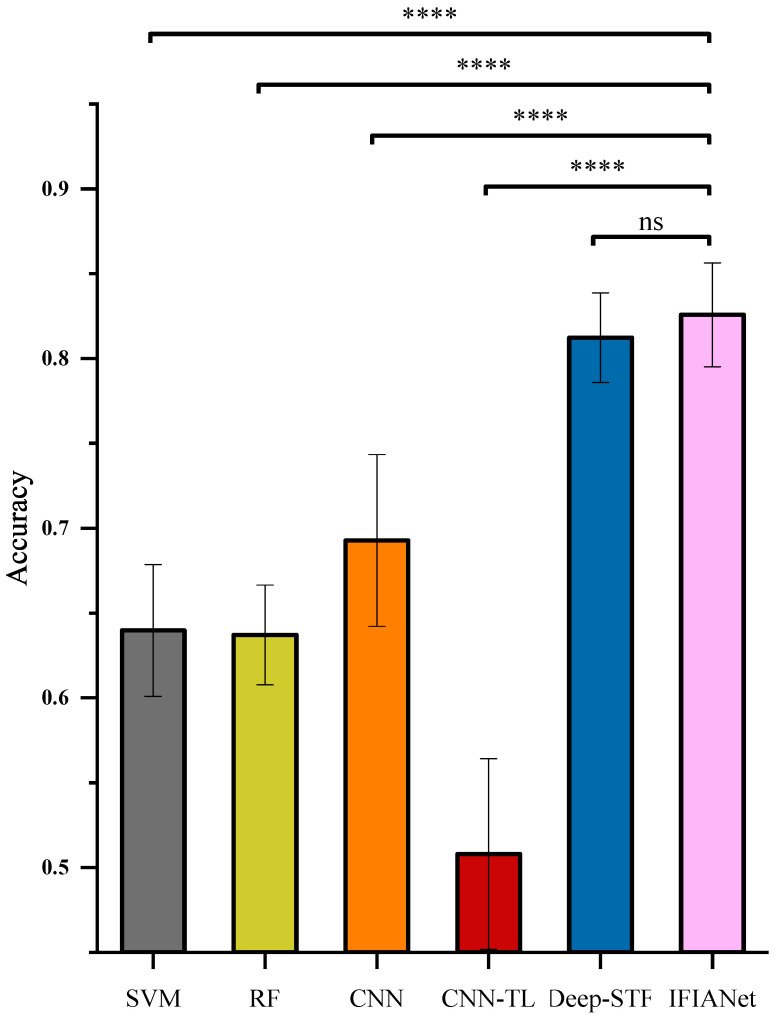
Comparison of classification performance. Statistical significance of model comparison results. Significance levels are based on Bonferroni-corrected *p*-values and are indicated as follows: **** p<0.0001; “ns” denotes no statistically significant difference between groups.

To evaluate the superiority of the IFIANet over existing methods, representative traditional machine learning approaches, including SVM [20] and RF [23], as well as three mainstream deep learning architectures, namely CNN [40], the CNN-LSTM-based Deep-STF [34], and CNN-TL(CNN–Transformer–LSTM) [32], were selected as comparative models. All models were evaluated on the sEMG-based gait recognition dataset under identical training and validation conditions. Figure 7 illustrates the classification accuracy results of each model under the condition of $P_{\mathrm{label}}=100\;\mathrm{ms}$. To further compare the performance differences among models, a one-way repeated-measures ANOVA was conducted to examine the effect of model architecture on classification performance.The results revealed a significant main effect of model architecture (F(5,40)=289.15, p<0.001, $\eta_{g}^{2}=0.892$), followed by post hoc paired t-tests with Bonferroni correction.

As illustrated in Figure 7, the average accuracies of the traditional machine learning methods SVM and RF were 63.98±3.89% and 63.71±2.94%, respectively. In contrast, the IFIANet achieved an accuracy of 82.58±3.06%, representing improvements of 18.6% and 18.87% over SVM and RF, respectively. For deep learning models, CNN, CNN-TL, and Deep-STF achieved accuracies of 69.28±5.06%, 50.8±5.61%, and 81.23±2.64%, respectively, while the IFIANet still outperformed them by 13.3%, 31.78%, and 1.35%. Post hoc paired *t*-tests with Bonferroni correction were performed to compare the proposed model with other approaches. The proposed method significantly outperformed SVM, RF, CNN, and CNN-TL (all Bonferroni-corrected p<0.0001). Although the comparison between the proposed model and Deep-STF did not reach statistical significance after Bonferroni correction (p=0.145), a large effect size was observed (Cohen’s d = 0.886). Overall, the IFIANet outperformed both traditional machine learning and mainstream deep learning models in terms of accuracy and stability, demonstrating its effectiveness and superiority in sEMG-based motion intent recognition tasks.

### 5.7. Accuracy of Different Prediction Time for Labeling

**Figure 8:**
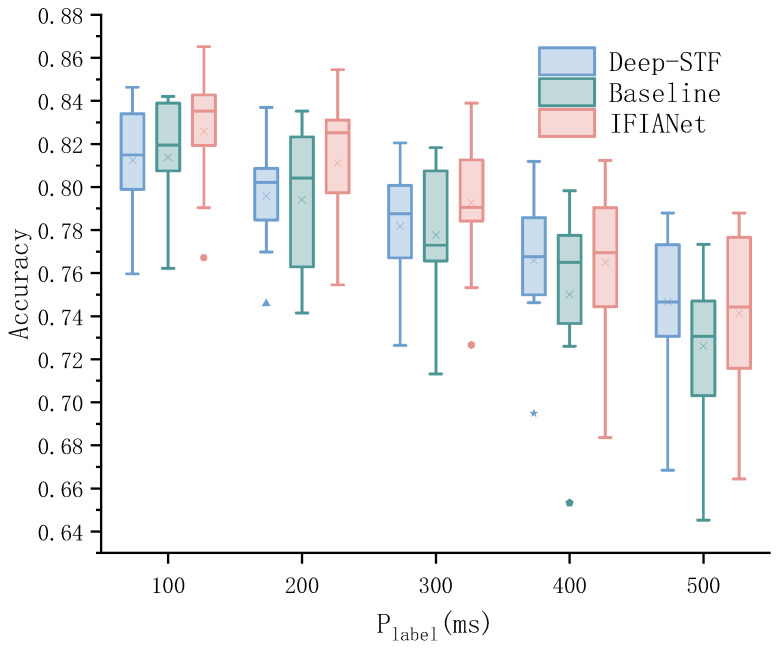
Model performance comparison across different prediction times.

We further investigated the impact of different prediction times on the recognition performance of each model, as shown in Figure 8. As the prediction time $P_{\mathrm{label}}$ increased from 100 ms to 500 ms, the recognition accuracy of the Deep-STF, baseline, and proposed models exhibited an overall decreasing trend. Taking the Deep-STF method as an example, when $P_{\mathrm{label}}$ was 100 ms, its maximum, upper quartile, median, lower quartile, and minimum values were 84.63%, 83.41%, 81.49%, 79.89%, and 75.97%, respectively. When $P_{\mathrm{label}}$ increased to 500 ms, these metrics decreased to 78.80%, 77.32%, 74.66%, 73.06%, and 66.85%, corresponding to reductions of 5.83%, 6.09%, 6.83%, 6.83%, and 2.91%, respectively. In addition, noticeable outliers were observed for Deep-STF at $P_{\mathrm{label}}$ values of 200 ms and 500 ms. In comparison, the baseline model slightly outperformed Deep-STF under shorter prediction-time conditions; however, when $P_{\mathrm{label}}$ increased to 300 ms, the overall performance of the baseline model became inferior to that of Deep-STF. For instance, when $P_{\mathrm{label}}$ was 100 ms, its maximum, upper quartile, median, lower quartile, and minimum values were 84.21%, 83.90%, 81.95%, 80.75%, and 76.22%, respectively. When $P_{\mathrm{label}}$ increased to 500 ms, these values declined to 77.33%, 74.70%, 73.06%, 70.31%, and 64.53%, corresponding to reductions of 6.88%, 9.20%, 8.89%, 10.44%, and 11.69%, respectively. Overall, the performance degradation of the baseline model was more pronounced than that of Deep-STF, and a clear outlier was observed at $P_{\mathrm{label}}=400$ ms.

Furthermore, incorporating the IFIA module into the baseline model resulted in a significant improvement in both recognition accuracy and prediction stability. Within the prediction-time range of 100–300 ms, IFIANet consistently achieved higher median and mean accuracies than the other models at all corresponding time points. As $P_{\mathrm{label}}$ increased from 100 ms to 500 ms, the median accuracy of IFIANet was 83.52%, 82.52%, 79.05%, 76.95%, and 74.43%, respectively, representing improvements of 1.57%, 2.10%, 1.75%, 0.45%, and 1.37% over the baseline model. Notably, the relative performance gain introduced by the IFIA module was the largest at $P_{\mathrm{label}}=$ 200 ms.

These results demonstrate that the IFIA module can consistently enhance model performance under different prediction-time conditions, exhibiting strong generalization capability. By effectively integrating frequency-domain and time–frequency-domain features, the module enables cross-domain adaptive feature interaction, thereby strengthening the model’s ability to discriminate movement intentions. Particularly under longer prediction times or more complex movement conditions, the IFIA module maintains stable recognition accuracy, validating its effectiveness in improving both model robustness and discriminative capability.

### 5.8. Influence of Inter-Subject Variability

To comprehensively validate the effectiveness of the IFIANet, we conducted a quantitative evaluation using commonly adopted classification metrics, including Accuracy, Precision, Recall, and F1-Score. The performance results of nine subjects on these four metrics are summarized in Table 6. As shown, under the condition of $P_{\mathrm{label}}=100\;\mathrm{ms}$, the model achieved average Accuracy, Precision, Recall, and F1-Score of 82.58%, 78.46%, 75.96%, and 76.91%, respectively. Except for Subjects 2 and 4, all other subjects achieved accuracies above 81%, demonstrating stable overall performance and indicating that the IFIANet exhibits good adaptability ability across individuals.

From the overall perspective of the four metrics, the model maintained high levels of Accuracy and Precision, suggesting strong discriminative capability in distinguishing different motion intent classes. Meanwhile, the Recall and F1-Score remained balanced, indicating reliable recognition performance not only for major classes but also for minority ones. From the perspective of inter-subject variability, the performance fluctuations mainly arise from physiological and experimental differences among subjects, such as muscle activation patterns, skin impedance, sensor adhesion position, and electrode spacing. These factors can influence the amplitude and spectral distribution of sEMG signals, leading to slightly lower recognition accuracy for certain individuals. Nevertheless, even in the presence of considerable inter-subject differences, the model still maintained high performance, demonstrating its ability to effectively extract subject-invariant time–frequency features and achieve strong robustness.

In addition, there exists a certain degree of class imbalance among different gait types in the dataset, particularly for the sitting, turning-around, and standing classes, which contain relatively fewer samples. This imbalance may further amplify the impact of inter-subject variability on the recall rate. Nevertheless, the IFIANet still outperforms traditional machine learning and mainstream deep learning approaches in terms of overall performance, demonstrating strong adaptability capability under conditions of complex individual differences and imbalanced sample distributions.

## 6. Discussion

In the field of lower limb exoskeleton control, achieving natural and coordinated human–robot collaboration remains a core objective. sEMG signals can reflect muscle neural activation prior to motion onset; thus, sEMG-based motion intention recognition has become a key approach for predicting human movement. However, effectively extracting and utilizing both temporal and spectral characteristics from sEMG signals remains a major challenge in this domain. This challenge depends not only on the quality of feature encoding but also on the model’s ability to represent complex dynamic patterns. Traditional sEMG recognition methods typically rely on handcrafted temporal, spectral, or time–frequency features combined with classical machine learning algorithms for classification. The performance of such approaches is highly dependent on feature selection and expert knowledge, resulting in limited generalization ability. With the development of deep learning, CNNs and their variants have been widely applied to automatic sEMG feature extraction, achieving remarkable progress in motion intention recognition. Nevertheless, sEMG signals exhibit strong nonstationarity and significant inter-subject variability. Furthermore, fixed sliding-window segmentation used during preprocessing may cut off or smooth short-term transient muscle activations, thereby weakening the model’s ability to capture instantaneous dynamics.

To address these issues, this study proposes the IFIANet framework, which employs a CNN–TCN structure for multi-scale time–frequency feature extraction and introduces the IFIA module to enhance global frequency-domain guidance. The CNN–TCN structure captures local dynamic patterns by modeling temporal dependencies across multiple frequency bands via frequency-division convolutions. Meanwhile, the IFIA module leverages global frequency-domain information extracted by FFT to dynamically weight and interactively fuse time–frequency features, compensating for potential spectral information loss during transformation and improving both feature completeness and discriminability. Experimental results on the MyPredict1 dataset—covering 17 types of gait intentions—demonstrate that the IFIANet achieves superior performance compared with traditional models. When $P_{\mathrm{label}}=100\;\mathrm{ms}$, the model attains an average recognition accuracy of 82.58%, representing a 1.19% improvement over the baseline model. Ablation studies further verify the effectiveness of the IFIA module in spectral feature compensation and time–frequency enhancement. In addition, to investigate the impact of prediction time on model performance, comparative experiments were conducted with $P_{\mathrm{label}}$ ranging from 100 ms to 500 ms. The results indicate that the IFIA module achieves its highest performance gain over the baseline model at a prediction time of 200 ms, with an improvement of 2.10%, demonstrating its effectiveness in providing robust discriminative capability and feature guidance. In within-subject experiments, the model achieved an average recognition accuracy exceeding 82% across nine participants, further demonstrating its strong robustness and generalization ability.

These findings demonstrate that the IFIA module effectively leverages global prior information from the frequency domain to guide the network in focusing on key spectral bands that may otherwise be neglected during time–frequency transformation. This enhances the model’s ability to distinguish subtle differences between static and dynamic gait patterns. The proposed frequency-guided mechanism not only overcomes the deficiency of CNN–TCN structures in spectral modeling but also provides a new perspective and methodological reference for time–frequency fusion learning based on sEMG signals.

Despite its strong performance in motion intention recognition, IFIANet still has several limitations. First, this study primarily focuses on steady-state gait prediction and does not explicitly model transitions between different gait phases; therefore, its adaptability to dynamic transitional scenarios remains to be further validated. Second, the use of lower limb exoskeletons can substantially alter gait patterns and muscle activation [41]. However, the proposed model has not been trained or evaluated under exoskeleton-assisted conditions, which limits its direct applicability to practical human–robot collaborative control. In addition, factors such as electrode displacement, muscle fatigue, and transient movements may degrade model performance, while sEMG signals with low signal-to-noise ratios (SNR) could further reduce prediction accuracy, thereby affecting the synchronization and control performance of exoskeleton assistance. Future work will explore the use of more diverse training data to further enhance the robustness and real-world applicability of the proposed model.

## 7. Conclusions

This study proposes an innovative gait intention recognition framework, IFIANet, designed to enhance the performance of lower limb exoskeleton human–machine interaction systems based on sEMG signals. First, the CWT and FFT are employed to map raw time-domain signals into the time–frequency and frequency domains, respectively, enabling multi-dimensional feature representation. Then, a CNN–TCN-based time–frequency feature learning network is constructed, which utilizes a frequency-wise convolution structure to effectively extract multi-scale time–frequency features. This design not only reduces model parameters but also preserves frequency-band independence and strengthens inter-frequency feature representation. On this basis, the IFIA module is introduced to leverage global frequency-domain information to guide time–frequency feature learning, compensating for potential spectral information loss during time–frequency transformation and thereby improving the completeness and robustness of feature representations.

Comprehensive ablation and comparative experiments were conducted on the public MyPredict1 dataset. The results demonstrate that the proposed method consistently outperforms other models across various gait intention recognition tasks, maintaining high accuracy and stability under different prediction times and within-subject conditions. In particular, the IFIA module exhibits sustained advantages in frequency-domain compensation and time–frequency feature enhancement, confirming its effectiveness and robustness in multi-scenario gait recognition.

## Figures and Tables

**Table 1:** Seventeen motion intentions included in the MyPredict1 dataset.

Label	Definition
1	Sitting
2	Standing
3	Walking
4	Turn around axes
5	Ascending stairs
6	Descending stairs
7	Ascending a ramp
8	Descending a ramp
9	Walking on uneven terrain
10	Diagonal step in front left
11	Diagonal step in front right
12	Diagonal step backwards right
13	Diagonal step backwards left
14	Small steps in front
15	Small steps to the right
16	Small steps backwards
17	Small steps to the left

**Table 2:** Convolutional layer architecture and parameter configuration.

Modules	Layers	Parameters	Output
Input	-	-	1×1200×8
Time–Frequency Block	Conv	3×1, 1	1×1198×8
	Avg Pooling	4×1, stride 4	1×299×8
	Conv	3×1, 1	1×297×8
	Avg Pooling	4×1, stride 4	1×74×8
	Conv	3×1, 1	1×72×8
	Avg Pooling	4×1, stride 4	1×18×8
	Aggregation	-	30×18×8
	Conv	3×1, 16	16×16×8
	BatchNorm	-	16×16×8
	Leaky-ReLU	0.01	16×16×8
	Flatten	-	16×128
Input	-	-	1×601×8
Frequency Block	Conv	3×1, 1	1×599×8
	Conv	3×1, 1	1×597×8
	Max Pooling	4×1, stride 4	1×149×8

**Table 3:** Number of parameters for each method.

Method	Baseline	Data-Level Fusion	Feature-Level Fusion	Decision-Level Fusion	IFIANet
Params	681,737	681,797	763,893	1,361,734	1,087,329

**Table 4:** Ablation study of the IFIA module.

Method	Accuracy (%)
Baseline	80.75
IFIA without Channel Attention	80.75
IFIA without Cross-Interaction	80.61
IFIA without Temporal Compression	80.02
IFIANet	81.59

**Table 5:** Ablation study on *r* in the IFIA module.

*r*	1	2	4	8
Accuracy (%)	81.12	81.27	81.59	80.61

**Table 6:** Performance of five-fold cross-validation evaluation.

Subject	Accuracy (%)	Precision (%)	Recall (%)	F1 (%)
1	81.94	76.86	73.70	74.38
2	79.04	72.66	70.75	71.54
3	83.52	76.96	74.65	75.60
4	76.72	73.12	70.30	71.44
5	83.63	81.54	77.61	79.00
6	84.28	83.64	81.38	82.40
7	86.52	84.05	83.00	83.38
8	85.29	78.20	74.67	76.14
9	82.29	79.18	77.57	78.31
Mean	82.58	78.46	75.96	76.91

## Data Availability

The data presented in this study are available on request from the corresponding author.

## Appendix A. IFIA Module Implementation Details

For implementation convenience, we do not explicitly indicate tensor reordering operations to maintain notational clarity. The structure is illustrated in Figure 3. Specifically, the model input consists of frequency-domain features ($X_{f}\in R^{1\times F_{f}\times C}$) and time–frequency features ($X_{tf}\in R^{F_{tf}\times T\times C}$), where $F_{f}$ denotes the sequence length in the frequency domain, *C* represents the number of channels, and $F_{tf}$ and *T* denote the sequence lengths along the frequency and temporal dimensions of the time–frequency features, respectively. First, global average pooling and global max pooling are applied to the $X_{f}$ to compress the spatial dimensions and extract channel-wise information, yielding the channel descriptor vectors ($Z_{\mathrm{avg}}\in R^{C\times 1\times 1}$, $Z_{\max}\in R^{C\times 1\times 1}$). Subsequently, both descriptors are passed through a shared-weight multilayer perceptron (MLP) that performs layer-by-layer nonlinear transformations and feature learning to obtain the channel attention representations ($A_{\mathrm{avg}}\in R^{C\times 1\times 1}$, $A_{\max}\in R^{C\times 1\times 1}$). The MLP consists of two fully connected layers and ReLU. To further emphasize frequency-domain channels with guiding significance, the two attention weights are concatenated and then passed through a 1×1 convolution to generate the fused attention weights ($A_{f}\in R^{C\times 1\times 1}$), followed by a Sigmoid activation to generate the final channel attention weights ($W_{f}\in R^{C\times 1\times 1}$). These weights are then multiplied with the original feature map to produce the weighted output (${\tilde{X}}_{tf}\in R^{F_{tf}\times F_{f}\times C}$). The process can be summarized as follows:

(A1) $$Z_{\mathrm{avg}}=f_{\mathrm{avg}}\left(X_{f}\right),\;Z_{\max}=f_{\max}\left(X_{f}\right)$$

(A2) $$A_{\mathrm{avg}}=W_{2}\delta \left(W_{1}Z_{\mathrm{avg}}\right),\;A_{\max}=W_{2}\delta \left(W_{1}Z_{\max}\right)$$

(A3) $$A_{f}=\mathrm{Conv}1\times 1\left(\left[A_{\mathrm{avg}};A_{\max}\right]\right)$$

(A4) $$W_{f}=\sigma \left(A_{f}\right)$$

(A5) $${\tilde{X}}_{tf}=W_{f}⊙X_{tf}$$

where $f_{\mathrm{avg}}$ and $f_{\max}$ denote the outputs of the global average pooling and global max pooling layers, respectively. $W_{1}\in R^{(C/r)\times C}$, $W_{2}\in R^{C\times (C/r)}$, and δ represent the weights of the two fully connected layers in the MLP and the ReLU, with *r* being the reduction ratio (set to 4 in this study). Conv1×1 denotes a 1×1 convolution operation, and σ denotes the sigmoid activation function.

After obtaining the channel attention, and prior to cross-domain interaction, to establish a correspondence with the frequency-domain features, to match the scale of the frequency-domain features, the time dimension of ${\tilde{X}}_{tf}$ is first compressed and normalized to obtain a time–frequency representation of corresponding scale. The specific procedure is as follows:

(A6) $${\bar{X}}_{tf}=\frac{1}{\sqrt{T}}\sum_{t=1}^{T}\left|{\tilde{X}}_{tf}\left(t\right)\right|$$

To achieve fine-grained interaction between frequency-domain and time–frequency features, the IFIA module independently performs a multi-head self-attention mechanism at the channel level [42]. At this stage, the input consists of *C* channels of frequency-domain ($X_{f}^{\left(c\right)}\in R^{F_{f}\times 1}$) and time–frequency features (${\bar{X}}_{tf}^{\left(c\right)}\in R^{F_{tf}\times 1}$). To project them into a unified embedding space, a one-dimensional convolutional projection layer is employed instead of the traditional linear mapping layer [43,44]. This design effectively captures local contextual dependencies, making it more suitable for modeling non-stationary sEMG signals. Subsequently, layer normalization (LayerNorm) is applied to standardize the input of each channel, generating the corresponding query ($Q^{\left(c\right)}\in R^{F_{tf}\times D}$), key ($K^{\left(c\right)}\in R^{F_{f}\times D}$), and value ($V^{\left(c\right)}\in R^{F_{f}\times D}$) representations. Based on these representations, multi-head attention weights ($A^{\left(c\right)}\in R^{F_{tf}\times F_{f}}$) are computed, and the outputs are then averaged along the feature dimension (dim = 1) to obtain the frequency-domain guided weights ($H^{\left(c\right)}\in R^{F_{tf}\times 1}$). The detailed computation process is shown as follows:

(A7) $$Q^{\left(c\right)}=\mathrm{LN}\left({\mathrm{Linear}}_{\mathrm{conv}}\left({\bar{X}}_{tf}^{\left(c\right)}\right)\right),\;K^{\left(c\right)},V^{\left(c\right)}=\mathrm{LN}\left({\mathrm{Linear}}_{\mathrm{conv}}\left(X_{f}^{\left(c\right)}\right)\right)$$

(A8) $$A^{\left(c\right)}=\sigma \left(\frac{Q^{\left(c\right)}{\left(K^{\left(c\right)}\right)}^{T}}{\sqrt{d_{k}}}\right)$$

(A9) $$H^{\left(c\right)}=f_{\mathrm{mean}}\left(A_{c}^{\left(c\right)}\right)$$

where ${\mathrm{Linear}}_{\mathrm{conv}}$ denotes a one-dimensional convolutional projection layer with a kernel size of 3, and LN represents LayerNorm.

The frequency-domain guidance weights are multiplied with the original time–frequency features to obtain frequency-modulated time–frequency features (${\hat{X}}_{tf}^{\left(c\right)}\in R^{1\times F_{\mathrm{tf}}}$) on a channel-by-channel basis to achieve frequency-guided energy modulation, as shown in Equation (A10). This process enables the time–frequency features to receive global spectral guidance and suppression along the frequency dimension, thereby enhancing their responsiveness to the dominant energy distributions.

(A10) $${\hat{X}}_{tf}^{\left(c\right)}=H^{\left(c\right)}⊙{\bar{X}}_{tf}^{\left(c\right)}$$

Subsequently, the original time–frequency features of each channel ($X_{tf}^{\left(c\right)}\in R^{T\times F_{tf}}$) are concatenated with the frequency-modulated time–frequency features after LayerNorm to obtain the query (${\hat{Q}}^{\left(c\right)}\in R^{\left(T+1\right)\times F_{tf}}$), key (${\hat{K}}^{\left(c\right)}\in R^{\left(T+1\right)\times F_{tf}}$) and value (${\hat{V}}^{\left(c\right)}\in R^{\left(T+1\right)\times F_{tf}}$) representations, followed by a multi-head attention operation to obtain the fused representation (${\hat{A}}^{\left(c\right)}\in R^{\left(T+1\right)\times F_{tf}}$). The first *T* time steps are selected to preserve the components corresponding to the original time–frequency structure and remove redundant sequences, resulting in the output (${\tilde{A}}^{\left(c\right)}\in R^{T\times F_{tf}}$). This process enables the frequency-domain guidance to adaptively enhance or suppress each temporal point within the time–frequency sequence, thereby achieving dynamic cross-domain feature fusion. The detailed process is expressed as follows:

(A11) $${\hat{Q}}^{\left(c\right)},{\hat{K}}^{\left(c\right)},{\hat{V}}^{\left(c\right)}=\left[\mathrm{LN}\left(X_{tf}^{\left(c\right)}\right);\mathrm{LN}\left({\hat{X}}_{tf}^{\left(c\right)}\right)\right]$$

(A12) $${\hat{A}}^{\left(c\right)}=\mathrm{LN}\left({\hat{Q}}^{\left(c\right)}+\sigma \left(\frac{{\hat{Q}}^{\left(c\right)}{\left({\hat{K}}^{\left(c\right)}\right)}^{T}}{\sqrt{d_{k}}}\right){\hat{V}}^{\left(c\right)}\right)$$

(A13) $${\tilde{A}}^{\left(c\right)}={\hat{A}}^{\left(c\right)}\left[:,T,:\right]$$

Finally, the outputs from all channels are concatenated to obtain frequency-guided time–frequency representations ($\tilde{A}\in R^{F_{\mathrm{tf}}\times T\times C}$). After completing the interaction between frequency-domain and time–frequency features, a learnable scaling coefficient (α) is applied to fuse the frequency-guided time–frequency representations with the $X_{tf}$ to obtain the final out ($out\in R^{F_{tf}\times T\times C}$), as shown in Equation (A14). This design integrates independent modeling at the channel level with attention-guided fusion at the feature level, which substantially enhances the discriminative capability and robustness of time–frequency representations, thereby leading to a significant improvement in overall performance across multi-class sEMG recognition tasks.

(A14) $$out=X_{t}f+\alpha \cdot \tilde{A}$$
